# CD47-Dependent Regulation of Immune Checkpoint Gene Expression and MYCN mRNA Splicing in Murine CD8 and Jurkat T Cells

**DOI:** 10.3390/ijms24032612

**Published:** 2023-01-30

**Authors:** Sukhbir Kaur, Duha Awad, Richard P. Finney, Thomas J. Meyer, Satya P. Singh, Margaret C. Cam, Baktiar O. Karim, Andrew C. Warner, David D. Roberts

**Affiliations:** 1 Laboratory of Pathology, Center for Cancer Research, National Cancer Institute, National Institutes of Health, Bethesda, MD 20892, USA; 2 CCR Collaborative Bioinformatics, Resource, Office of Science and Technology Resources, National Cancer Institute, National Institutes of Health, Bethesda, MD 20892, USA; 3 Inflammation Biology Section, Laboratory of Molecular Immunology, National Institute of Allergy and Infectious Diseases, National Institutes of Health, Bethesda, MD 20892, USA; 4 Molecular Histopathology Laboratory, Laboratory Animal Sciences Program, Frederick National Laboratory for Cancer Research, National Cancer Institute, National Institutes of Health, Frederick, MD 21702, USA

**Keywords:** adaptive immunity, tumor microenvironment, immune checkpoints, CD47, thrombospondin-1, n-Myc, alternative splicing, cytotoxic T cells

## Abstract

Elevated expression of CD47 in some cancers is associated with poor survival related to its function as an innate immune checkpoint when expressed on tumor cells. In contrast, elevated CD47 expression in cutaneous melanomas is associated with improved survival. Previous studies implicated protective functions of CD47 expressed by immune cells in the melanoma tumor microenvironment. RNA sequencing analysis of responses induced by CD3 and CD28 engagement on wild type and CD47-deficient Jurkat T lymphoblast cells identified additional regulators of T cell function that were also CD47-dependent in mouse CD8 T cells. MYCN mRNA expression was upregulated in CD47-deficient cells but downregulated in CD47-deficient cells following activation. CD47 also regulated alternative splicing that produces two N-MYC isoforms. The CD47 ligand thrombospondin-1 inhibited expression of these MYCN mRNA isoforms, as well as induction of the oncogenic decoy MYCN opposite strand (MYCNOS) RNA during T cell activation. Analysis of mRNA expression data for melanomas in The Cancer Genome Atlas identified a significant coexpression of MYCN with CD47 and known regulators of CD8 T cell function. Thrombospondin-1 inhibited the induction of TIGIT, CD40LG, and MCL1 mRNAs following T cell activation in vitro. Increased mRNA expression of these T cell transcripts and MYCN in melanomas was associated with improved overall survival.

## 1. Introduction

The identification of signaling pathways that limit the activation of innate and adaptive immune responses and therapeutics that block these pathways, known as immune checkpoint inhibitors (ICI), has resulted in dramatic improvements in long-term survival for some cancers [1]. ICI targeting the PD-1 and CTLA4 immune checkpoints on CD8 T cells or their counter-receptors have become the standard of care for treating immunogenic cancer, such as cutaneous melanoma, but many patients fail to respond [2]. This has encouraged efforts to identify additional immune checkpoints and new ICI that could expand the number of patients and cancer types that benefit from ICI therapy. 

Thrombospondin-1 (TSP1) is a secreted matricellular protein that inhibits T cell activation by engaging its receptor CD47 [3,4,5,6,7,8]. Antibody blockade or antisense knockdown of CD47 increases T cell receptor-dependent killing of tumor cells by CD8 T cells in vitro and in vivo [9,10]. Antisense knockdown of CD47 can delay the growth of some cancers in immune-competent mice, but tumor ablation and increased long-term survival have been achieved only by combining with local tumor irradiation, cytotoxic chemotherapy, or anti-CTLA4 or anti-PD-1 ICI [8,9,10,11,12]. 

In addition to its function as an adaptive immune checkpoint on T cells, elevated CD47 expression on tumor cells can serve as an innate immune checkpoint by engaging its counter receptor signal regulatory protein-α (SIRPα), which is expressed on macrophages, neutrophils, dendritic cells, and activated NK cells [13,14,15]. Humanized antibodies and other biologics that serve as decoys to target the CD47/SIRPα pathway have entered human clinical trials, and anecdotal evidence for efficacy has been reported in phase 1 trials when combined with other targeted therapies [16,17,18,19,20]. 

To further develop ICI targeting CD47, we need to better understand the functions of its interactions with TSP1 and SIRPα in the tumor microenvironment. Although the loss of either CD47 or TSP1 in the tumor microenvironment greatly enhanced tumor ablation by ionizing radiation in several immune-competent cancer models [10,21], we also found that, in the absence of tumor irradiation, B16 melanomas grew moderately faster in *cd47^−/−^* mice [22]. This was associated with transcriptome alterations, indicating that the absence of CD47 leads to increased exhaustion of NK cells and CD8 T cells in tumor-bearing *cd47^−/−^* mice [22,23]. This may involve the effects of CD47 signaling in immune cells on metabolism and integrin signaling pathways required for T cell differentiation, activation, and migration [5,24,25,26]. In addition, we found changes in the expression of immune checkpoints, including PD-1 and TIGIT on *cd47^−/−^* CD8 cells in the tumor microenvironment that could impair their function and suggest crosstalk between CD47 signaling and other immune checkpoint pathways [23]. 

Here, we have applied RNAseq analysis to further define CD47-dependent transcriptome changes during T cell activation in vitro and examined the potential relevance of these findings using cBioPortal tools to examine correlations between CD47-dependent gene expression and survival in cutaneous melanomas in The Cancer Genome Atlas (TCGA). 

## 2. Results

### 2.1. CD47 Regulates mRNA Expression of T Cell Activation Markers in Jurkat T Lymphoblasts

**Figure 1:**
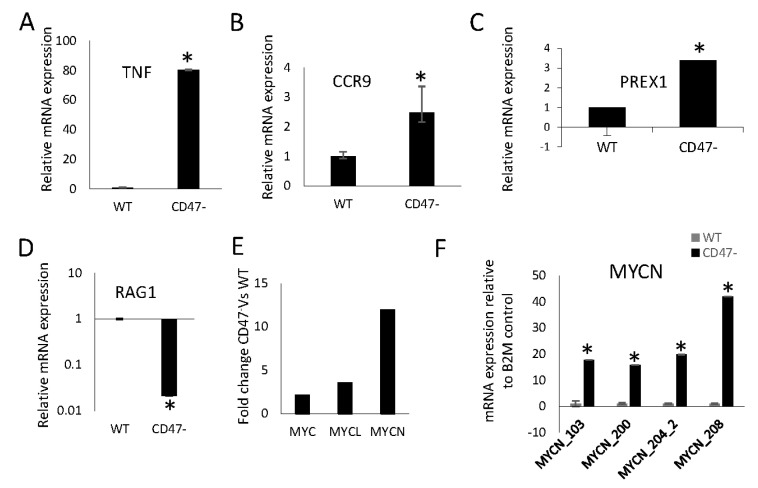
Validation of CD47-dependent genes identified by RNAseq in Jurkat T lymphoblasts. WT and CD47^−^ Jurkat T cells were activated on immobilized CD3 and CD28 antibodies and treated in the presence or absence of TSP1 for the indicated times. (**A**–**D**) Validation of differentially expressed genes (Supplementary Figure S1D), *TNF*, *CCR9*, *PREX*1, and *RAG*1 using WT and CD47^−^ Jurkat T cells via real-time PCR using B2M as the control. (**E**) Fold change in MYC, MYCN, and MYCL between WT and CD47^−^ Jurkat T cells via RNA sequencing (Supplementary Data S1). (**F**) Relative expression of MYCN mRNA determined using real-time PCR between WT and CD47- Jurkat T lymphoblasts. Error bars indicate technical replicates (* = *p*-values ≤ 0.05, *n* = 3).

Our RNAseq analysis of mouse CD8 T cells identified 199 CD47-dependent genes that are involved in CD8 cell responses to viral infection in mice [23,27]. To identify additional CD47-dependent genes, RNA sequencing was performed using WT and CD47^−^ Jurkat T lymphoblasts and analyzed using the NIDAP platform (GEO accession number GSE218256). We identified transcripts that differ basally between WT and CD47^−^ cells (Supplementary Figure S1D) and transcripts that respond differently to the engagement of CD3 and CD28 in the presence or absence of TSP1. (Supplementary Figure S1, Data S1). TNF, CCR9, PREX1, and RAG1 were selected as representative up and downregulated genes between CD47^−^ and WT Jurkat T cells, and were validated using quantitative real-time PCR (Figure 1A–D). 

### 2.2. CD47 Preferentially Regulates Expression of the MYC Gene Family Member MYCN in Mouse CD8 T Cells

Consistent with our previous observations of a CD47-dependent enrichment of an MYC target signature in mouse CD8 T cells [23] and CD47-dependent regulation of MYC expression in mouse CD8 T cells and Jurkat T lymphoblasts [28], MYC mRNA in the RNAseq was increased 2.1-fold in CD47^− ^ relative to WT Jurkat cells (Figure 1E and Supplementary Data S1). However, the other two members of the *MYC* gene family, MYCN (12-fold) and MYCL (3.5-fold) were also elevated in CD47^−^ Jurkat cells relative to WT. Increased MYCN expression was confirmed via real-time PCR analysis using four sets of MYCN primers (Supplementary Data S3, Figure 1F). 

**Figure 2:**
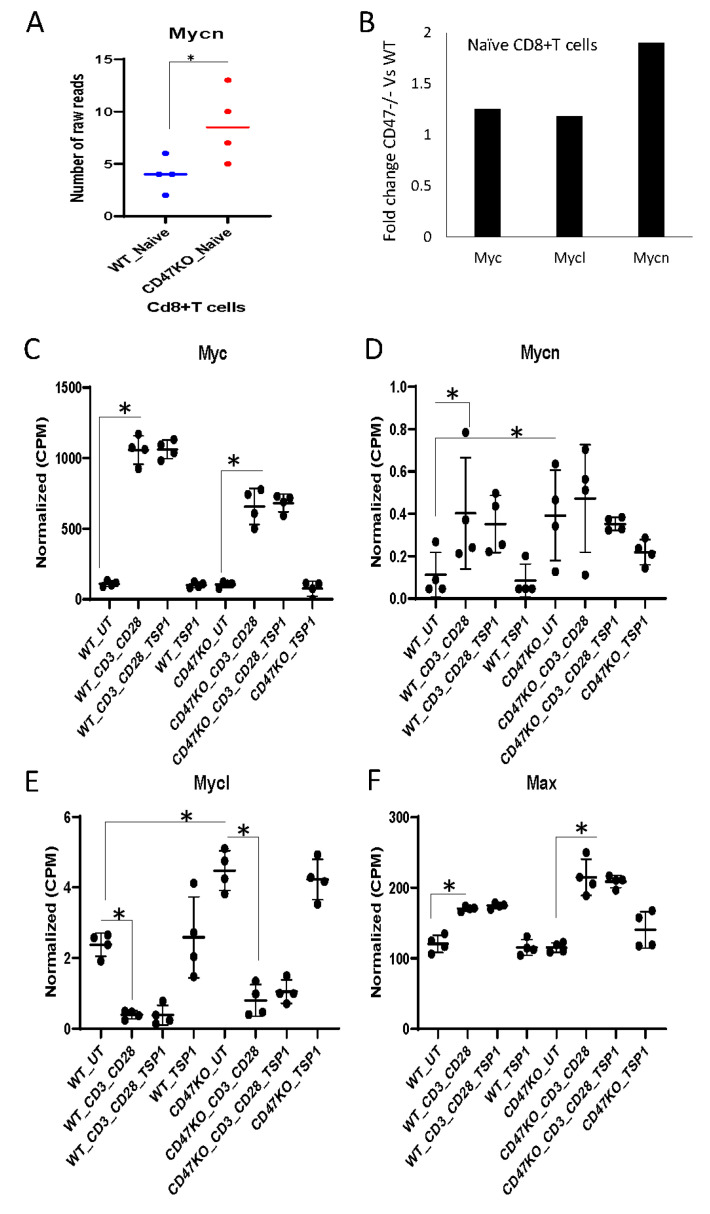
CD47-dependent regulation of Myc family genes by activation and TSP1 in mouse CD8^+^ T cells. (**A**) Expression of Mycn mRNA in naïve WT and *cd47^−/−^* mouse CD8+ T cells. (**B**) Fold change of selected Myc family genes (Myc, Mycn, and Mycl) in *cd47^−/−^* as compared to naïve WT mice (unpublished). WT CD8^+^ T cells extracted from RNA sequencing raw reads. (**C**–**F**) mRNA expression of Myc, Mycn, Mycl, and Max (normalized CPM) from WT and *cd47^−/−^* CD8^+^ T cells that were activated with anti-CD3 plus anti-CD28 antibodies and treated in the presence or absence of TSP1 for 6 h. Error bars indicate independent biological replicates (* = *p*-values ≤ 0.05, *n* = 4).

Correspondingly, Mycn mRNA expression was significantly higher in naïve *cd47^−/−^* mouse CD8^+^ T cells compared to WT cells (Supplementary Data S4, Figure 2A). Myc and Mycl mRNA levels were only modestly increased in *cd47^−/−^* as compared to WT mouse CD8^+^ T cells (Figure 2B). We further examined the effects of TCR signaling and TSP1 on the expression of c-Myc family genes using RNA sequencing data of CD8^+^ T cells derived from WT and *cd47^−/−^* mouse spleens [23] (Supplementary Data S4). As expected, Myc mRNA was strongly induced following activation on immobilized anti-CD3 and CD28 for 6 h compared to CD8^+^ T cells cultured in RPMI medium with 2% FCS for 6 h, but the induction was lower in *cd47^−/−^* CD8^+^ T cells (Figure 2C). Treatment with 1 μg/mL (2.2 nM) TSP1 did not affect MYC mRNA levels in the absence or presence of activation. Consistent with these results, we previously reported reduced Myc mRNA in positively selected CD8 T cells from the spleen of *cd47^−/−^* mice [28]. Basal Mycn and Mycl mRNA expression was higher in *cd47^−/−^* CD8^+^ T cells cultured in RPMI medium with 2% FCS for 6 h than in the corresponding WT CD8^+^ T cells (Figure 2D,E). Activation increased Mycn mRNA in WT but not in *cd47^−/−^* CD8^+^ T cells (Figure 2D). This response was not sensitive to TSP1. In contrast to Myc and Mycn, Mycl mRNA levels were suppressed in activated WT and *cd47^−/−^* CD8^+^ T cells (Figure 2E). The c-Myc binding protein Max [29] was not differentially expressed between untreated WT and *cd47^−/−^* CD8^+^ T cells and moderately induced following activation (Figure 2F). 

### 2.3. CD47 Regulates Expression of MYCN Splice Variants

**Figure 3:**
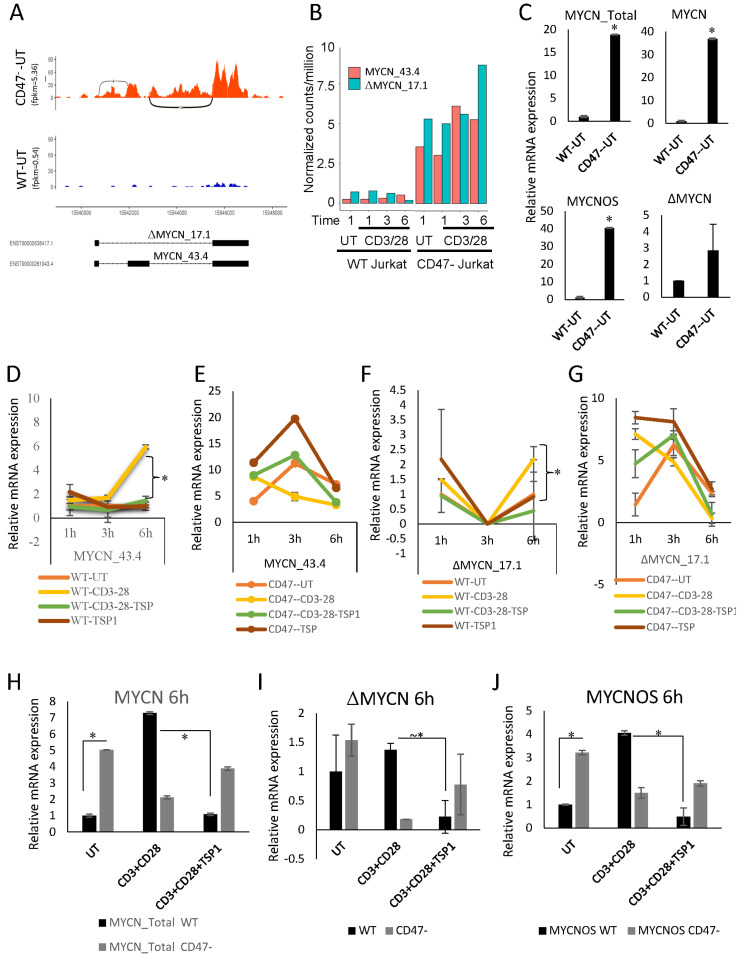
CD47-dependent regulation of MYCN splice variants. (**A**) Sequence coverage for unspliced and two isoforms, ENST00000281043.4 (MYCN_43.4) and ENST00000638417.1(ΔMYCN_17.1), in unstimulated WT and CD47^−^ Jurkat cells. (**B**) Normalized CPM for MYCN 43.4 and ΔMYCN_17.1 isoforms in untreated (UT) WT and CD47^−^ Jurkat cells and cells activated on anti-CD3 plus CD28 for the indicated times. (**C**) MYCN isoform mRNA expression in untreated Jurkat cells determined by real-time PCR using specific primers normalized to B2M, * = *p*-values ≤ 0.05. (**D–G**) mRNA expression of MYCN splice isoforms (common MYCN_43.4 and ΔMYCN_17.1) during WT and CD47^−^ Jurkat cell activation using CD3 plus CD28 antibodies in the presence or absence of 1 μg/mL TSP1 for 1, 3, and 6 h via real-time PCR. 18S ribosomal RNA primers (*n* = 2, replicates) were used as the control for normalization. Error bars indicate technical replicates for total MYCN and ΔMYCN (* = *p*-values ≤ 0.05, *n* = 3). (**H**–**J**) Jurkat (JK) and CD47^−^ JinB8 cells were plated on anti-CD3 and CD28 coated plates in the presence or absence of 1 μg/mL TSP1 for 6 h. Relative expression of ΔMYCN, MYCNOS, and total MYCN at 6 h was normalized to 18S (* = *p*-values ≤ 0.05, *n* = 3, technical replicates).

MYCN mRNA undergoes alternative splicing, and the antisense transcript MYCNOS [30] and the splice variant ΔMYCN [31] have been studied in early development [32]. RNAseq data suggested that MYCN isoform ENST00000281043.4 MYCN-201 (MYCN 43.4) is the predominant isoform expressed in CD47^−^ Jurkat cells, but the low expression limited interpretation of splicing in untreated WT Jurkat cells (Figure 3A, Supplementary Figure S2). The alternatively spliced isoform ENST00000638417.1 MYCN-202 (ΔMYCN 17.1) was not sufficiently expressed in Jurkat cells for the RNAseq analysis to detect potential changes following activation, but the normalized CPM suggested some induction of both isoforms following 1 to 6 h activation of CD47^−^ cells (Figure 3B). Therefore, real time PCR using published primers [33] was used to quantify expression of MYCNOS, ΔMYCN and MYCN in WT and CD47^−^ Jurkat T cells (Figure 3C). The PCR analysis showed that full-length MYCN 43.4 is responsible for most of the ~18-fold increase in MYCN_total in untreated CD47^−^ cells compared to WT Jurkat T cells. ΔMYCN 17.1 was elevated 2.9-fold in CD47^−^ cells compared to WT Jurkat T cells, and MYCNOS expression was also significantly higher in untreated CD47^−^ cells (Figure 3C). These results confirmed the induction of full-length MYCN 43.4 following 6 h activation in WT cells.

Although the RNAseq data did not show a significant effect of TSP1 on the activation-induced increase in total MYCN mRNA abundance, a physiological concentration of TSP1 (2.2 nM) inhibited the induction of MYCN 43.4 following 6 h activation in WT cells without having any effect in the absence of activation (Figure 3D). In contrast, treatment of CD47^−^ cells with TSP1 alone moderately increased MYCN 43.4 at all time points, but the expression fell from 1 to 6 h with activation, and TSP1 did not inhibit the increased MYCN 43.4 levels in activated CD47^−^ cells (Figure 3E). The low expression of ΔMYCN 17.1 limited its analysis, but TSP1 significantly inhibited the activation-induced increase at 6 h in WT cells (Figure 3F). No consistent trends or effects of TSP1 were observed for ΔMYCN 17.1 in CD47^−^ cells (Figure 3G). 

Regulation of MYCNOS mRNA by activation and TSP1/CD47 signaling was examined in a second real-time PCR analysis (Figure 3H–J). Controls using primers to detect total MYCN established that the inhibition of activation-induced mRNA expression at 6 h by 2.2 nM TSP1 occurred only in cells expressing CD47 (Figure 3H). Consistent with Figure 3F, TSP1 also inhibited mRNA for the ΔMYCN 17.1 isoform following activation, but only in Jurkat cells expressing CD47 (Figure 3I). MYCNOS RNA was also induced at 6 h of activation and inhibited by TSP1 in WT Jurkat cells (Figure 3J). In contrast, the higher basal expression of MYCNOS in the untreated CD47^−^ cells at 6 h was decreased in cells plated on the activating antibodies, and TSP1 did not alter this expression. Therefore, the regulation of MYCNOS RNA expression induced by T cell activation by TSP1 is also CD47-dependent.

Multicolor fluorescent in situ hybridization (FISH) using RNAscope® technology was used to examine the potential heterogeneity of MYCN isoform expression in WT versus CD47^−^ Jurkat cells and the induction of specific isoforms following activation. To determine whether the percentage of cells expressing specific MYCN isoforms changed during TCR stimulation in the presence or absence of TSP1, treated and control WT and CD47^−^ Jurkat T cells were fixed, and expression of MYCN 43.4 (red) and ΔMYCN 17.1 (yellow) was detected, as shown for representative fields in Supplementary Figure S3B with nuclear DAPI staining. The percentage of positive cells for two splice variants is shown in Supplementary Figure S3C (Data S2A,B). The percent cells positive for the predominant isoform MYCN 43.4 did not differ significantly between WT and CD47^−^ Jurkat T cells (Supplementary Figure S3C,D). As expected, TCR stimulation moderately increased the percentage of cells positive for both isoforms, but TSP1 treatment showed only modest effects on the percent positive cells. This suggested that TSP1 and TCR activation generally regulates MYCN 43.4 and ΔMYCN 17.1 mRNA expression rather than inducing expression by a specific subset of cells. 

### 2.4. High Expression of CD8 T Cell Markers, CD47 Dependent Immune Regulators, and MYCN Correlates with Improved Patient Survival in Cutaneous Melanoma

**Figure 4:**
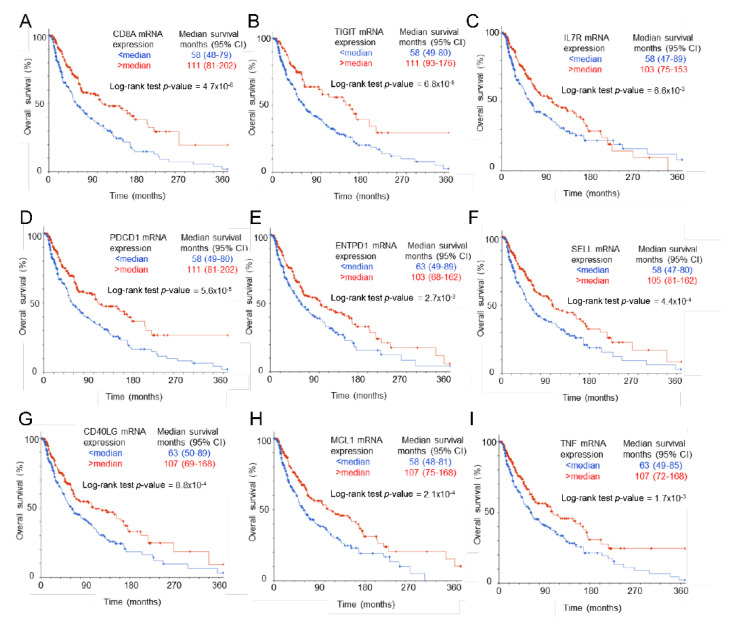
Association of CD8 T cell marker and CD47-dependent regulators of T cell function with overall survival in human melanomas. Kaplan–Meier plots for overall survival of 469 TCGA cutaneous melanoma patients with higher versus lower CD8 T cell infiltration indicated by CD8A mRNA expression greater than or less than the median (**A**) or with mRNA expression greater than or less than the median of the CD47-dependent genes TIGIT (**B**), IL7R (**C**), PDCD1 (PD-1, **D**), ENTPD1 (CD39, **E**) SELL (CD62L, **F**), CD40LG (**G**), MCL1 (**H**), and TNF (**I**). The median survival of each group is presented with the respective 95% confidence intervals.

The increased growth of B16 melanomas in *cd47^−/−^* mice relative to the growth of the same tumors in WT mice is associated with impaired CD8 T cell and natural killer cell functions in the tumor microenvironment [22,23]. The improved overall and disease-free survival of cutaneous melanoma patients with elevated CD47 mRNA expression in their tumors [22] is consistent with these data and supports a protective role for CD47 in the melanoma tumor microenvironment that is inconsistent with the prevailing hypothesis that CD47 functions primarily on cancer cells to shield them from clearance by innate immune cells [34,35]. In addition to the previously reported increase in NK early effector markers and improved survival in melanomas with higher CD47 [22], further analysis of the melanoma TCGA data showed an improved overall survival of patients bearing tumors with higher CD8 T cell infiltration, indicated by CD8A mRNA expression (111 versus 58 months, log-rank *P* = 4.7 × 10^−6^, Figure 4A). Analysis of TCGA RNAseq data from cutaneous melanomas established that mRNAs encoding the CD8 T cell coreceptor subunits CD8α and CD8β have strong positive correlations with CD47 mRNA expression (Table 1). 

Our previous study identified several regulators of CD8 T cell function for which protein expression was regulated by loss of CD47 expression in the tumor microenvironment, including TIGIT, PD-1, CD39, CD62L, and CD127 [23]. Higher mRNA expression for each of the corresponding mRNAs in TCGA melanomas was associated with significantly improved overall survival (Figure 4B–F). Expression of the regulators of T cell function CD40 ligand, TNF, and MCL1, which were identified herein as CD47-dependent, showed similar significant associations with improved overall survival (Figure 4G–I). Analysis of RNAseq data from the TCGA melanomas established that mRNAs encoding the CD8 T cell markers CD8a and CD8b, the CD47-dependent T cell activation markers CD69 and TNFα, and all of the above regulators of CD8 T cell function have strong positive correlations with CD47 mRNA expression (Table 1). 

**Figure 5:**
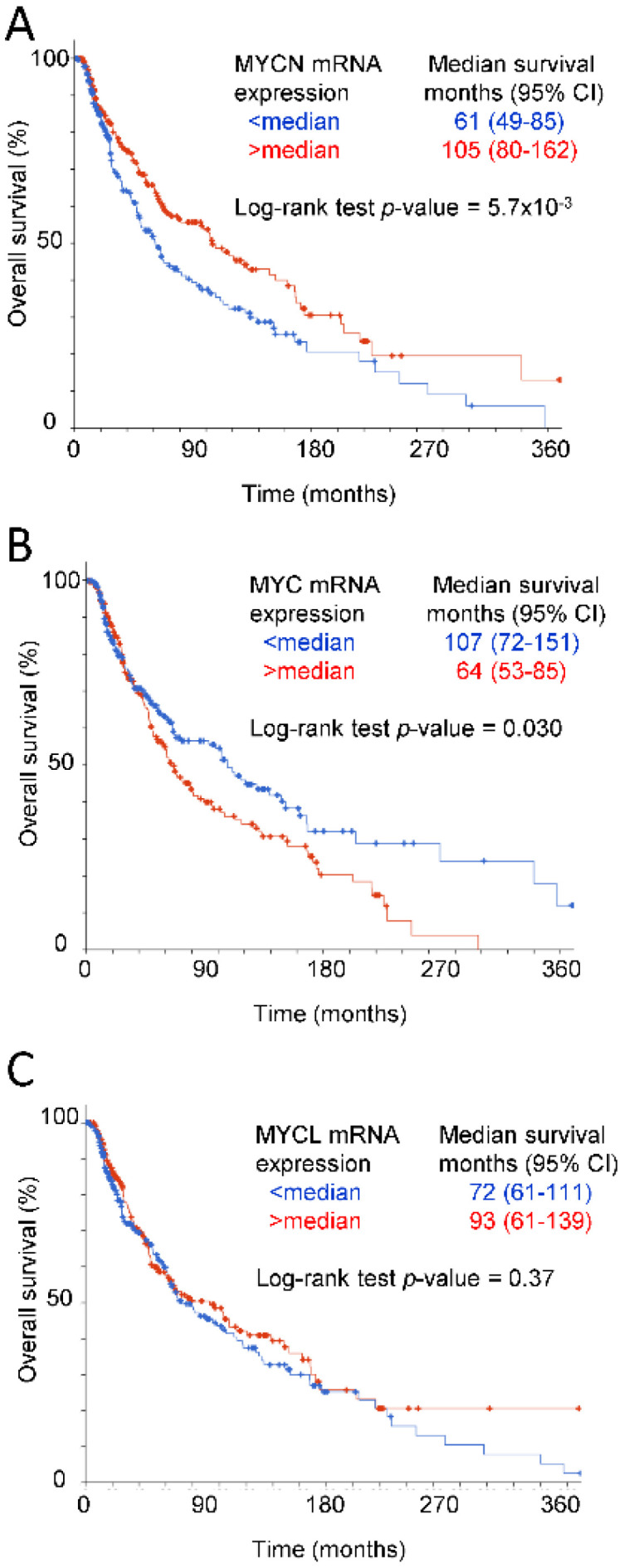
Association of MYC family mRNA expression with overall survival in human melanomas. Kaplan–Meier plots indicate the overall survival of 469 melanoma patients with MYCN (**A**), MYC (**B**), or MYCL (**C**) mRNA expression greater than or less than the median. The median survival of each group is presented with the respective 95% confidence intervals.

The MYC paralog MYCN also showed a significant positive correlation between elevated mRNA expression and improved overall survival (105 versus 61 months, *p* = 5.7 × 10^−3^, Figure 5A), whereas mRNA expression of the paralog MYCL was not associated with overall survival (Figure 5C). In contrast, elevated MYC mRNA had a marginally significant negative correlation with overall survival (64 versus 107 months, log-rank *p* = 0.030, Figure 5B). Consistent with these data, MYCN mRNA expression was positively correlated in the TCGA melanomas with mRNA expression for the CD8 T cell markers, activation markers, and functional regulators, including CD47, whereas MYC mRNA exhibited consistent negative correlations with mRNA expression for the same genes (Table 1).

We also considered the alternate hypothesis that the effects of MYCN or MYC expression on survival results from their expression in melanoma cells. Analysis of TCGA RNAseq data for melanoma cell lines in The Cancer Cell Line Encyclopedia [37] indicated minimal or undetectable MYCN expression in melanoma cells (Supplementary Figure S4). To further address this, we examined NCR3LG1, an established MYC-induced target in melanoma cells, and an MYCN-induced target in neuroblastoma cells [36], but found no significant coexpression (MYCN vs. NCR3LG1 −0.026 *p* = 0.57; MYC vs. NCR3LG1 0.067 *p* = 0.15, Table 1). Therefore, the correlation between MYCN and survival probably relates to a function of MYCN in nonmalignant cells in the melanoma microenvironment, but we cannot exclude additional roles for MYCN expressed in stromal cells other than T cells.

TCGA data for additional cancers with RNAseq and survival data were surveyed to determine whether the MYCN correlations identified for cutaneous melanoma extend to other tumor types (Table 2). Expression of the mRNAs encoding the CD8 coreceptor subunits and the CD47-dependent activation markers CD69 and TNFα was positively correlated with mRNA expression of MYCN in head and neck squamous cell carcinoma, breast invasive carcinoma, prostate carcinoma, and lung squamous cell carcinoma, but no significant correlations between MYCN mRNA and overall survival were observed for these cancers. Additional cancers examined showed either no correlation or negative correlations between MYCN and the T cell mRNAs and there was no correlation with survival, except in the case of renal clear cell carcinomas. The latter association appears to be T cell-independent, but may relate to the identification of MYCN in a prognostic signature for renal clear cell carcinoma [38].

Most of these cancers lack significant MYCN expression (Supplementary Figure S4), but MYCN is an oncogenic driver in pediatric neuroblastomas [39]. Therefore, neuroblastomas are a negative control to exclude regulation of T cells in the tumor microenvironment by MYCN expression in malignant cells. We examined MYCN mRNA coexpression with T cell function markers and CD47 in these tumors (Supplementary Table S3). In contrast to the melanoma tumor data in Table 1, MYCN showed minimal coexpression with T cell markers and functional genes in pediatric neuroblastomas. Likewise, CD47 showed minimal coexpression with the same genes. Furthermore, pediatric neuroblastomas diverged from melanomas in that MYC mRNA showed strong positive rather than negative correlations with the same genes. Therefore, MYC but not MYCN or CD47 expression in neuroblastoma cells appears to be a significant regulator of T cell function in the tumor microenvironment.

### 2.5. TSP1 Inhibits CD47-Dependent Induction of MCL1, CD40 Ligand, and TIGIT in T Cells

**Figure 6:**
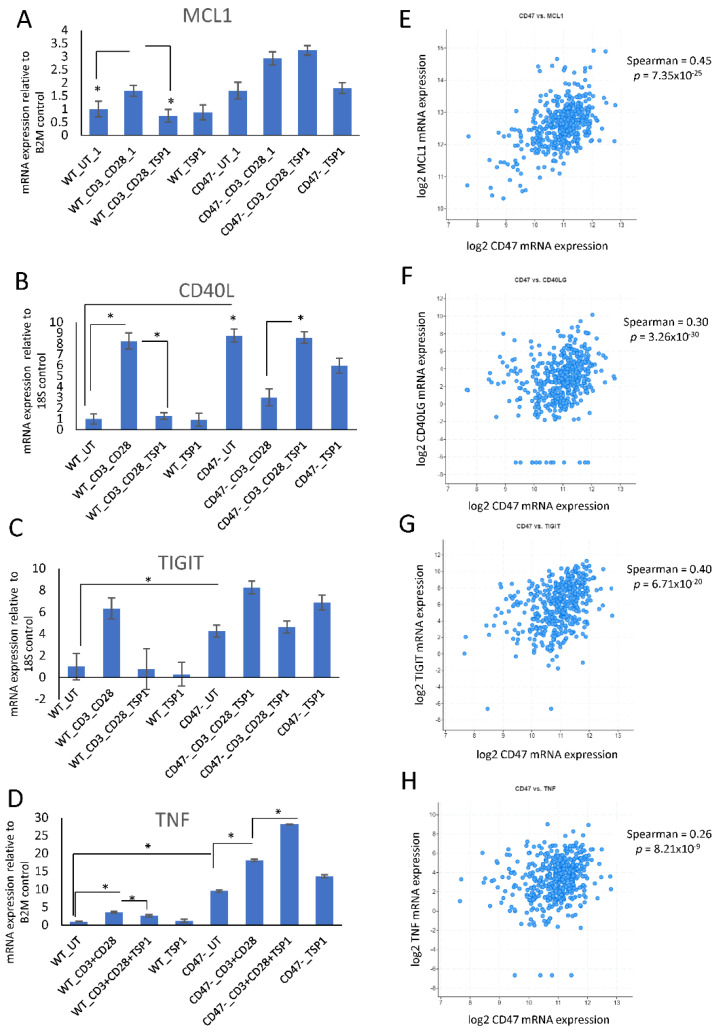
CD47 dependence of immune checkpoint and MCL1mRNA expression in vitro and in melanomas. (**A–C**) WT and CD47^−^ Jurkat T cells were activated on wells coated with anti-CD3 plus anti-CD28 in the presence or absence of 1 μg/mL TSP1 for 6 h. Total RNA was extracted using the Trizol method, and mRNA expression of MCL1 (**A**), CD40L (**B**), TIGIT (**C**), and TNF (**D**) was determined via real-time PCR normalized to 18S or B2M controls, as indicated. Error bars indicate technical replicates for MCL1, CD40, and TIGIT (* = *p*-values ≤ 0.05, *n* = 3). (**E**–**H**) Coexpression of CD47 mRNA with MCL1 (**E**), CD40LG (**F**), TIGIT (**G**), and TNF (**H**) mRNAs was analyzed for 472 TCGA cutaneous melanoma samples.

Based on their strong positive correlations with CD47 mRNA expression in the TCGA melanoma data (Figure 6D–F, Table 1), we selected MCL1, CD40LG, TNF, and TIGIT to examine their regulation by CD47 signaling in human Jurkat T lymphoblast cells. Basal expression of MCL1 mRNA did not differ significantly between WT and CD47^−^ Jurkat cells (Figure 6A), whereas CD40 ligand, TNF, and TIGIT mRNAs were basally elevated in the CD47^−^ cells (Figure 6B–D). Activation using immobilized anti-CD3 plus anti-CD28 for 6 h increased MCL1, and 2.2 nM TSP1 inhibited this induction without altering expression in unstimulated cells (Figure 6A). MCL1 was also increased following activation of CD47^−^ cells, but TSP1 did not inhibit this induction, demonstrating CD47-dependence for this inhibitory activity of TSP1. In WT cells, induction of the CD40 ligand, TIGIT and TNF mRNAs and TSP1 inhibition of that induction was also observed (Figure 6B–D). In contrast, the high basal CD40 ligand mRNA in CD47^−^ cells was reduced following TCR stimulation, and TSP1 remarkably reversed that inhibition. We have previously reported CD47-independent effects of TSP1 on other T cell genes, including CD69 [4,40], EEF1A1, ETS1, and YOD1 [41], but the relevant TSP1 signaling receptor remains to be defined. Similarly, TSP1 had a modest inhibitory effect on the induction of TIGIT mRNA in the CD47^−^ cells, which is also presumably CD47-independent. Consistent with these in vitro data, CD47 mRNA expression in TCGA melanomas had highly significant positive correlations with MCL1, CD40LG, TIGIT, and TNF mRNA expression (Figure 6E–H, Table 1). The expression of these genes was also positively correlated with that of MYCN in the melanomas but inversely correlated with that of MYC (Table 1).

## 3. Discussion

MYCN is expressed during human and murine embryonic development [42,43]. In adults, MYCN expression persists in early hematopoietic stem cells but is lost following their differentiation into lineage-committed cells [44]. MYCN expression increased when hematopoietic stem cells were exposed to stress. MYCN has been widely studied as an oncogene that is amplified in pediatric neuroblastoma [45], but it is also expressed in some B cell lymphomas [46], a subset of T cell acute lymphoblastic leukemias (T-ALL) [47], erythroleukemias [48], and leukemia cells lines including Jurkat T lymphoblasts [49,50]. To our knowledge, however, MYCN expression and function have not previously been documented in mature T cells. Here, we identified a splice variant of MYCN that is induced in activated CD8 T cells, which is inhibited by TSP1 in a CD47-dependent manner, and found that MYCN expression positively correlates with multiple markers of CD8 T cells and their activation, including CD8A, CD8B, TNF, TIGIT, CD40LG, IL7R, PDCD1 (PD-1), SELL (CD62L), ENTPD1 (CD39), MCL1, and CD47 in cutaneous melanomas. MYCN induction in T cells coincides with the induction of MCL1, TIGIT, TNF, and CD40LG following activation, which is also inhibited by TSP1. Taken together, these data suggest that the MYCN mRNA detected in the TCGA melanomas is expressed in CD8 T cells and is another marker of their activation, which would be consistent with the positive correlation between MYCN and overall survival. Determining whether specific isoforms of MYCN play direct roles in the CD47-dependent regulation of other immune regulators in CD8 cells in vitro or in melanomas will require further study. 

TIGIT is upregulated following T cell activation and functions as an immune checkpoint to limit antitumor immunity [51]. Loss of CD47 in mice bearing B16 melanomas increased the abundance of TIGIT+ CD8 T cells and NK cells in the spleen while reducing CD8 T cell numbers and the percent of TIGIT+ CD8 T cells in tumors [22,23]. In this context, a recently reported dual inhibitor of CD47/SIRPα and TIGIT/PVR pathways merits further study for utility as an ICI in melanoma [52]. In contrast to the association of high CD47 and TIGIT with increased survival in melanoma, it is notable that the opposite was recently reported for lung squamous cell carcinoma [53]. This is consistent with the disconnect between increased expression of CD8 T cell markers and survival in the TCGA data.

MCL1 is a BCL2 family anti-apoptotic protein that was identified in the Jurkat RNAseq data as CD47-dependent and confirmed by qPCR. The association of higher MCL1 with high CD47 and improved survival in melanomas suggests that this increased MCL1 is expressed by immune cells in the tumor microenvironment. MCL1 mRNA was induced by activation of WT and CD47^−^ Jurkat cells, but the expression was inhibited by TSP1 only in WT cells that express CD47. A previous study found that MCL1 protein levels were increased in Jurkat cells treated with a CD47 antibody that induced cell death and inhibits SIRPα binding [54]. Jurkat cells do not express SIRPα, so the mechanism for this response is unclear. Another study using aptamers that knock down CD47 and MCL1 suggested additive immune-stimulating effects when combined in a mouse triple-negative breast cancer model [55]. Alternatively, spliced MCL1 transcripts can either increase or decrease apoptosis [56]. 

We previously identified MYC as a target of CD47 signaling that is elevated in CD47^−^ Jurkat cells and in cells and tissues from *cd47^−/−^* mice, but notably not elevated in *cd47^−/−^* CD8 T cells [28]. TSP1 inhibited MYC expression in Jurkat cells in a CD47-dependent manner [28]. MYC is an important oncogene and a universal amplifier of gene expression [57], but it also regulates the expression of immune checkpoints, such as CD47 and PD-L1 [58]. Thus, MYC supports both the growth of cancer cells and their evasion of antitumor immunity [59]. Myc is also induced during lymphocyte activation, and our recent study of WT versus *cd47^−/−^* CD8 T cell activation showed gene enrichment of hallmarks of Myc targets [23]. Although Mycn can replace the Myc functional role in murine lymphocyte development and activation [60], the present data indicates that MYC and MYCN have opposing relationships with the expression of T cell markers and functional genes in cutaneous melanomas. In both Jurkat cells and CD8 T cells, we found MYCN upregulated more strongly than MYC in the absence of CD47. The increased Mycn may compensate for the lower Myc expression we previously reported in naïve *cd47^−/−^* mouse CD8+ T cells [28]. 

TSP1 has multiple activities in the tumor microenvironment that modulate tumor growth and metastasis via CD47-dependent and -independent mechanisms [8,61]. Conversely, CD47 functions in the tumor microenvironment can depend on the engagement of either SIRPα or TSP1. Recent efforts to exploit CD47 as a therapeutic target have assumed a primary role in the SIRPα-dependent “don’t eat me” function of CD47 expressed in malignant cells. The direct effects of antibodies or decoys that block this interaction include enhancement of macrophage and neutrophil clearance of tumor cells and increased antigen presentation to CD8 T cells [14,62]. 

Contrary to the proposed primary role for innate immune cells in the anticancer activities of CD47-targeted therapeutics, several independent groups have found CD8 T cells to be essential for therapeutic responses to CD47 blockade in immune-competent mouse tumor models including for cutaneous melanoma [10,63,64,65]. Furthermore, a therapeutic blockade of CD47 by antibodies or suppression of CD47 expression is sufficient to enhance the direct TCR-dependent killing of tumor cells by mouse or human CD8 T cells [9,10]. Correspondingly, blockade of TSP1 signaling or loss of CD47 expression on T cells increases their acute activation and TCR signaling [3,4,5,6,7,23]. 

In addition to increased CD8A and CD8B mRNA indicating that melanomas with elevated CD47 contain more CD8 T cells, the present data correlating CD47 with higher TIGIT, CD40LG, PD-1, TNF, and the antiapoptotic MCL1 suggests these intratumoral CD8 cells are more active and resistant to apoptotic death. On the other hand, sustained expression of the immune checkpoints TIGIT and PD-1 can lead to T cell exhaustion and resistance to immunotherapy, which is consistent with the moderate impairment of the antitumor and antiviral functions of NK and CD8 T cells in *cd47^−/−^* mice [22,23,66]. Because B16 melanomas growing in either *cd47^−/−^* or *thbs1^−/−^* mice were more sensitive to ablation by tumor irradiation [10,21], which required CD8 T cells [10], the increased expression of damage-associated molecular patterns (DAMPs) induced by ionizing radiation may overcome the moderate impairment *cd47^−/−^* T cells. Thus, partial therapeutic blockade of CD47 signaling can be beneficial even though the complete loss of CD47 impairs NK and CD8 T cell functions. The cross-regulation we demonstrate between CD47 and other immune checkpoints we identified in melanomas supports further therapeutic trials combining CD47 blockades with ICIs targeting PD-1 and TIGIT. 

In addition to its relevance to melanoma, the present data may provide insights into overcoming treatment resistance for the four cancers identified in Table 2, where CD47-dependent T cell activation and function markers were positively correlated with the presence of CD8 T cells in tumors but not with survival. In the case of breast carcinoma, the disconnect between intratumoral CD8 T cell numbers and their antitumor function has been linked to immunosuppression by Tregs [67]. Although the 4T1 breast carcinoma model is not optimal for studying adaptive antitumor immunity, the synergy between CD47 blockade and chemotherapy response in this model suggests the relevance of CD47 as a therapeutic target for overcoming treatment resistance in breast cancer [11].

## 4. Materials and Methods

### 4.1. Cell Culture and T Cell Activation Assays for mRNA Sequencing 

WT and CD47^−^ Jurkat T cells were cultured routinely, as described earlier ([28]. CD3 Monoclonal Antibody (OKT3), eBioscience™ and anti-CD28 (CD28 Monoclonal Antibody (CD28.2), Functional Grade, eBioscience™ were purchased from Thermo Fisher Life Technologies Corporation, Waltham, MA USA. For the T cell activation assay, 10 cm plates (BD, Biosciences, San Jose, CA USA) were coated with 2 μg/mL of anti-CD3 and 4 μg/mL of anti-CD28 antibodies in PBS at 4 °C overnight. Approximately 5 × 10^6^ cells in 5 mL were plated for 1 h, 3 h, and 6 h for each treatment using HITES medium [4], and 1 μg/mL of TSP1 was added either alone or in combination with anti-CD3 and anti-CD28 antibodies. Cell pellets from each treatment were washed with PBS, and total RNA was extracted using TriPURE. The concentration of RNA and RNA quality was measured via Nanodrop. Flow cytometry confirmed that the CD47- mutant lacks cell surface CD47 expression and that CD47 expression in the WT cells was slightly increased following activation on anti-CD3 and anti-CD28 antibodies (Supplementary Figure S3D). 

Mouse primary CD8^+^ T cells were obtained from mouse spleens by negative selection, as described previously [23,28], using the mouse CD8a+ T Cell Isolation Kit (Miltenyi Biotec, Gaithersburg, MD, USA). The freshly isolated CD8^+^ T cells were plated on anti-mouse CD3- and CD28-coated antibodies for 6 h in the presence or absence of TSP1 (1 μg/mL) using RPM1 medium with 2% FCS. The RNA was extracted, and mRNA sequencing was performed, as described earlier [23]. Total raw reads from each replicate (n = 4) were averaged and graphed with standard error using Graph pad Prism 8.

### 4.2. Bulk mRNA Sequencing and Analysis 

All samples were processed with the TruSeq mRNA kit using ~40M PE125 reads per sample. The RNA-Seek pipeline (https://github.com/CCBR/RNA-seek) was used to process reads. Briefly, reads were trimmed for adapters and low-quality bases using the Cutadapt v1.18 [68] before alignment to the hg38 reference genome (GRCh38) and transcripts using STAR v2.4.2a in 2-pass mode [69]. Gene and isoform expression was quantified using RSEM v1.3.3 [70] with GENCODE annotation version 36 [71].

Downstream analysis and visualization were performed within the NIH Integrated Analysis Platform (NIDAP) using R programs developed on Palantir Foundry (Palantir Technologies, Denver, CO, USA). Expected counts from RSEM for both genes and isoforms were imported into the NIH Integrated Data Analysis Platform (Palantir Technologies) for downstream analysis. Briefly, low-count genes were filtered prior to CPM and quantile normalization using Limma voom [72,73], followed by differential expression of genes analysis. Batch correction was performed using the ComBat function of the sva [74]. Sashimi plots of MYCN isoform coverage were generated using ggsashimi [75]. 

Raw RSEM counts of mouse WT and CD47KO CD8^+^T cells from [23], and with the indicated treatments with or without TSP1 (n = 4 biological replicates) were filtered using one Minimum Number of Samples with Nonzero Counts in a Group and normalized with the “Quantile” method using the NIDAP platform, as indicated above. The *p*-values were calculated using DEG lists from the following comparisons: CD47KO_UT vs. WT_UT, WT_CD3_CD28 vs. WT_UT, WT_CD3_CD28_TSP1 Vs WT_CD3_CD28, WT_TSP1 vs. WT_UT, CD47KO_CD3_CD28 vs. CD47KO_UT, CD47KO_CD3_CD28_TSP1 vs. CD47KO_CD3_CD28, and CD47KO_TSP1 vs. CD47KO_UT. The logCPM values of Myc, Mycn, Mycl, and Max transcripts from the DEG list were converted into CPM and plotted in Figure 2C–F using GraphPad Prism. 

### 4.3. Real-Time PCR Analysis

Jurkat WT and JinB8 CD47^−^ T cells were cultured routinely, as described earlier [4,76]. For a T cell activation assay, 12-well plates were coated with anti-human-CD3 (2 μg/mL, Clone OKT3,) and anti-human CD28 (4 μg/mL) in PBS at 4°C overnight. An amount of ~1 × 10^6^/mL cells using 2% FBS (Gibco, Billings, MT, USA) and RPMI-1640 medium (Thermo Fischer Scientific, Waltham, MA, USA) were plated for the indicated times. TSP1 (1 μg/mL) was added either alone or in combination with anti-CD3 plus anti-CD28-coated antibodies, and untreated were used as the control. After indicated times, each cell pellet/treatment was harvested, centrifuged, and washed with PBS. The total RNA extractions were performed using TriZol, Qiazol, or Tri-pure reagents. The concentration of RNA and quality was measured via Nanodrop, and real-time PCR was performed as described earlier [41,77], using the listed primers (Supplementary Table S1). MYCN (NM_005378.5) primers were designed using primer blast NCBI, as shown in Supplementary Data S3, the primer sequence for MYCN splice variant/isoforms. Total MYCN, MYCNOS, ΔMYCN, and MYCN primers were used [33].

### 4.4. TCGA Data Analysis

Cutaneous melanoma data (TCGA) for samples with mRNA expression with z-scores relative to diploid samples (RNA Seq V2 RSEM) were analyzed using cBioPortal tools [78,79] to determine mRNA coexpression. A median cutoff was used to segregate samples with high versus low expression of the indicated genes for examining relationships between mRNA expression and overall survival.

### 4.5. Fluorescent In Situ Hybridization (FISH)

WT and CD47^−^ cells were cultured routinely, as described earlier. For a T cell activation assay, 60 cm^3^ plates were coated with 2 μg/mL of anti-CD3 (CD3 Monoclonal Antibody (OKT3), eBioscience™) and 4 μg/mL of anti-CD28 (CD28 Monoclonal Antibody (CD28.2), Functional Grade, eBioscience™) at 4C overnight. An amount of 1 × 10^6^/ mL cells (25 mL/25 million cells) in 2% FBS media were plated. A total of 1 μg/mL of TSP1 was added either in the combination of anti-CD3 plus anti-CD28 or alone for 6 h. The cells were harvested, and pellets were made using the Cell Harvest Protocol (Pathology Histology Lab). The small fractions of WT and CD47^−^ Jurkat cells were analyzed for flow cytometry analysis before sending them to the core facility, as shown in Supplementary Figure S3D. An RNAscope® LS 2.5 Probe, Hs-MYCN, homo sapiens v-myc avian myelocytomatosis viraloncogene neuroblastoma-derived homolog (common MYCN_43.4) mRNA ENST00000281043.4, and BA-Hs-MYCN-202-E1E2 targeting 152-188 of ENST00000638417.1 (ΔMYCN_17.1 mRNA) probe was designed. ΔMYCN_17.1 probes (ENST00000638417.1) do not cross-react with MYCN_43.4 (ENST00000281043.4).

The expression of *Homo sapiens* ΔMYCN_17.1 was detected by staining 5 μm FFPE tissue sections with a BaseScope™ 2.5 LS Probe BA-Hs-MYCN-202-E1E2 (Advanced Cell Diagnostics, Inc., Newark, CA, USA (ACD), Cat# 1189948-C1) with the BaseScope™ LS Reagent Kit-RED (ACD, Cat# 323600) using the Bond RX auto-stainer (Leica Biosystems, Deer Park, IL, USA) with a tissue pretreatment of 15 minutes at 95 °C with Bond Epitope Retrieval Solution 2 (Leica Biosystems, Deer Park, IL, USA), and 25 minutes of Protease III (ACD) at 40 °C. The Basecope™ LS Probe BA-DapB-1ZZ (Bacillus subtilis dihydrodipico-linate reductase (dapB) gene, Cat# 701028, was used as a negative control. The Basecope™ LS Probe BA-Hs-PPIB-1zz (Cat# 710178) was used as a technical control to ensure the RNA quality of tissue sections was suitable for BaseScope staining.

The expression of common MYCN_43.4 was detected with the RNAscope® 2.5 LS Probe, Hs-MYCN-C1 (ACD, Cat# 417508), using the RNAscope LS Multiplex Fluorescent Assay (ACD, Cat# 322800) and a 1:750 dilution of TSA-Cyanine 5 Plus (Akoya Biosciences, Marlborough, MA, USA), was sequentially stained on the same slide as the Basescope with a Bond RX auto-stainer. The RNAscope 2.5 LS Negative Control Probe-DapB (Cat# 312038) was used as a negative control. The RNAscope® 2.5 LS Probe-Hs-PPIB (Cat# 313908) was used as a technical control to ensure the RNA quality of tissue sections was suitable for RNAscope staining. Slides were digitally imaged using an Aperio ScanScope FL Scanner (Leica Biosystems).

All image analysis was performed using HALO imaging analysis software (v3.4.2986.246; Indica Labs, Corrales, NM), and image annotations were performed by one pathologist (BK). The analysis was performed using FISH V3.1.3 in HALO to determine the percent positive cells. Areas of artifacts, such as folds and tears, were excluded from the analysis.

### 4.6. Statistical Analysis

ANOVA two-factor with replication was used for real-time PCR analysis. A *p*-value ≤ 0.05 is used as significant. Briefly, raw Ct values of reference controls, 18S or B2M, were subtracted from the respective mRNA Ct, and two-way ANOVA analysis with replicates was performed in Excel. The default parameters were set to make multiple comparisons based on treatments between Jurkat CD47- vs. Jurkat WT and CD47KO_CD8^+^ vs. WT CD8^+^T_cells. Significant *p*-values (≤0.05) for the sample or columns were indicated as asterisks (*). For Figure 3C, a t-test with equal variance was used. For Figure 3H–J, a t-test was performed using the default setting of CFX Maestro™ software with control untreated (WT) vs. CD3+CD28, while CD3+CD28+TSP1 vs. CD3+CD28 treatments were analyzed via ANOVA two-factor with replication. ~* is used for 0.052.

## Figures and Tables

**Table 1:** Coexpression of CD8 T cell markers and T cell regulatory genes with CD47, MYCN, and MYC mRNAs in TCGA skin cutaneous melanomas with RNA Seq V2 RSEM data was analyzed using cBioPortal tools (*n* = 472).

	Coexpression with CD47		Coexpression with MYCN^c^		Coexpression with MYC^b, c^	
Gene	Spearman’s Correlation	*p*-Value	Spearman’s Correlation	*p*-Value	Spearman’s Correlation	*p*-Value
CD8A ^c^	0.36	1.8 × 10^−15^	0.18	5.7 × 10^−5^	−0.16	3.3 × 10^−4^
CD8B	0.33	1.4 × 10^−13^	0.21	6.6 × 10^−6^	−0.29	7.8 × 10^−11^
CD69	0.50	2.5 × 10^−31^	0.24	9.6 × 10^−8^	−0.27	3.6 × 10^−9^
TNF	0.26	8.2 × 10^−9^	0.25	6.4 × 10^−8^	−0.24	8.2 × 10^−8^
TIGIT ^a^	0.40	6.7 × 10^−20^	0.20	1.7 × 10^−5^	−0.28	1.0 × 10^−9^
CD40LG ^c^	0.30	3.3 × 10^−11^	0.21	5.0 × 10^−6^	−0.28	1.0 × 10^−9^
IL7R (CD127) ^a, b, c^	0.39	2.2 × 10^−18^	0.23	6.4 × 10^−7^	−0.25	3.0 × 10^−8^
PDCD1 (PD-1) ^a^	0.31	7.5 × 10^−12^	0.20	1.7 × 10^−5^	−0.22	1.8 × 10^−6^
SELL (CD62L) ^a, b, c^	0.37	8.7 × 10^−17^	0.19	4.6 × 10^−5^	−0.27	1.6 × 10^−9^
ENTPD1 (CD39)^a^	0.27	1.6 × 10^−9^	0.20	1.2 × 10^−5^	−0.29	7.1 × 10^−11^
MCL1 ^c^	0.45	7.4 × 10^−25^	0.19	4.6 × 10^−5^	−0.22	9.5 × 10^−7^
CD47 ^c^	-	-	0.18	5.7 × 10^−5^	−0.16	3.3 × 10^−4^
NCR3LG1 ^d^	−0.01	0.76	−0.03	0.57	0.07	0.15

^a^ CD47-dependence in CD8 T cells was established in WT versus *cd47^−/−^* mice bearing B16 melanomas [22]. ^b^ Differentially regulated by activation in WT and *cd47^−/−^* CD8 T cells in vitro (RNAseq data from [23]). ^c^ Differentially expressed in Jurkat WT versus *CD47^−^* JinB8 cells (Supplementary Table S2, extracted from All_DEGs _pval05_ Data A). ^d^ Known MYC-induced gene in melanoma and other cancers [36].

**Table 2:** MYCN mRNA correlations with mRNA expression for CD8 T cell and activation markers and overall survival in TCGA cancer datasets.

Cancer	Spearman’s Correlation, *p*-Values				Log-Rank Correlation, *p*-Values
	CD8A	CD8B	CD69	TNF	Overall Survival
Cutaneous melanoma	+, 6.6 × 10^−6^	+, 1.7 × 10^−5^	+, 9.6 × 10^−8^	+, 6.4 × 10^−8^	+, 5.7 × 10^−3^
Head and neck SCC	+, 7.0 × 10^−11^	+, 3.2 × 10^−15^	+, 1.2 × 10^−16^	+, 0.12	NS
Breast invasive carcinoma	+, 4.5 × 10^−11^	+, 5.6 × 10^−14^	+, 6.4 × 10^−5^	+, 1.7 × 10^−3^	NS
Prostate adenocarcinoma	+, 2.2 × 10^−9^	+, 3.9 × 10^−3^	+, 1.3 × 10^−4^	+, 5.8 × 10^−11^	NS
Lung squamous cell carcinoma	+, 2.4 × 10^−3^	+, 8.4 × 10^−8^	+, 3.1 × 10^−5^	+, 1.7 × 10^−14^	NS
Lung adenocarcinoma	−, 1.9 × 10^−3^	NS	−, 7.8 × 10^−6^	NS	NS
Bladder urothelial carcinoma	−, 1.6 × 10^−5^	−, 1.9 × 10^−5^	−, 2.4 × 10^−4^	NS	NS
Papillary thyroid carcinoma	NS	−, 0.039	−, 6.7 × 10^−4^	NS	NS
Stomach adenocarcinoma	NS	NS	NS	NS	NS
Colorectal adenocarcinoma	NS	NS	NS	NS	NS
Kidney renal clear cell	NS	NS	−, 5.0 × 10^−4^	+, 1.8 × 10^−3^	+, 4.0 × 10^−3^
Renal papillary cell carcinoma	NS	NS	−, 0.045	NS	NS
Hepatocellular carcinoma	NS	NS	NS	+, 0.040	NS
Pancreatic adenocarcinoma	NS	NS	NS	NS	NS
Ovarian serous cystadenocarcinoma	NS	NS	−, 2.1 × 10^−4^	NS	NS
Uterine endometrial carcinoma	NS	NS	NS	NS	NS
Pediatric neuroblastoma	NS	NS	−, 2.3 × 10^−5^	NS	NS

## Data Availability

RNA sequencing data of Jurkat WT and Jurkat CD47^−^ anti-CD3+CD28 stimulated in the presence or absence of TSP1 time course 1, 3, and 6 hrs is available in the Gene Expression Omnibus (GEO) database https://www.ncbi.nlm.nih.gov/geo/query/acc.cgi?acc=GSE218256, GSE218256. All original code has been deposited at Github and is publicly available as of the date of publication at https://github.com/NIDAP-Community/CD47-dependent-regulation-of-immune-checkpoint-gene-expression-and-MYCN-mRNA-splicing.

## Supplementary Materials

The following supporting information can be downloaded at https://www.mdpi.com/article/10.3390/ijms24032612/s1, Supplementary Figures S1–S4: Data S1; Data S2; Data S3; Data S4. Supplementary Table S1: Primers.
