# Supplementary material for: CD47-Dependent Regulation of Immune Checkpoint Gene Expression and MYCN mRNA Splicing in Murine CD8 and Jurkat T Cells
Source: Int J Mol Sci. 2023 Jan 30;24(3):2612. doi: 10.3390/ijms24032612 (PMC9916813; doi:10.3390/ijms24032612)
Supplement: Supplementary file 1 [file ijms-24-02612-s001.zip › supplements/Kaur et al supplement 1-12-2023.pdf]

CD47-dependent regulation of immune checkpoint gene expression and MYCN mRNA splicing in murine CD8 and Jurkat T cells

Kaur et al.

Supplementary Figure Legends:

Figure S1. A-D) Graphic representation of flow chart for T cell activation experiment using anti-CD3 + anti-CD28 antibodies and treated in the presence or absence of TSP1 time course 1, 3 & 6 hrs. mRNA sequencing and its downstream QC analysis, PCA, Volcano plots. E-G) Different isoforms of MYCN, MYC and MYCL expressed in WT and CD47- cells.

Figure S2. A&B) Total MYCN and splice isoform coverage from RNAseq analysis of WT and $CD47^{-}$ Jurkat T cells with and without TCR stimulation using anti-CD3+CD28 in the presence or absence of TSP1 treatment.

Figure S3. A) Primers used for detecting specific MYCN splice isoforms and the antisense MYCNOS. B) WT and $CD47^{-}$ Jurkat cells were plated on anti-CD3 plus anti-CD28 coated plates in the presence or absence of 1 µg/ml TSP1 for 6 h. The cells were pelleted and fixed using FFPE Cell Embedding Protocol with thrombin and fibrinogen (MCL-histo). The fixed pellets were sectioned, stained for Common MYCN_ 43.4 _ (Red), ΔMYCN_17.1 (YFP) and DAPI (Blue). Representative 50x50 µm fields are shown from the full images in Data S2B. C). The number of total positive MYCN_43.4 cells are analyzed using HALO imaging analysis software. D) The WT and $CD47^{-}$ T cells were validated for CD47 surface staining via flow cytometry.

Figure S4. Analysis using cBioPortal tools of MYCN mRNA expression TCGA RNAseq data for 1156 cancer cell lines in The Cancer Cell Line Encyclopedia (Ghandi et al. 2019). Data are grouped by cancer type and MYCN expression is normalized as RKPM.

Supplementary Table S3 Coexpression of CD8 T cell markers and functional genes with CD47, MYCN and MYC mRNAs in TCGA Pediatric neuroblastomas RNA Seq V2 RSEM data analyzed using cBioPortal tools (n=143).

Gene	Coexpression with CD47		Coexpression with MYCN[c]		Coexpression with MYC[b, c]	
	Spearman's correlation	p-value	Spearman's correlation	p-value	Spearman's correlation	p-value
CD8A[c]	0.18	0.03	-0.03	0.70	0.12	0.16
CD8B	0.18	0.03	-0.08	0.34	0.22	$7.2x10^{-3}$
CD69	0.10	0.23	-0.34	$2.3x10^{-5}$	0.59	$5.2x10^{-15}$
TNF	ND		ND		ND	
TIGIT[a]	0.09	0.27	-0.18	0.03	0.39	$2.0x10^{-6}$
CD40LG[c]	0.21	0.01	-0.12	0.15	0.26	$1.6x10^{-3}$
IL7R (CD127)[a, b, c]	0.21	0.01	-0.17	0.04	0.38	$3.5x10^{-6}$
PDCD1 (PD-1)[a]	0.09	0.30	-0.18	0.03	0.43	$9.9x10^{-8}$
SELL (CD62L)[a, b, c]	0.21	0.01	-0.15	0.06	0.37	$5.4x10^{-6}$
ENTPD1 (CD39)[a]	-0.09	0.30	-0.23	$6.4x10^{-3}$	0.33	$6.6x10^{-5}$
MCL1[c]	-0.10	0.25	-0.38	$2.7x10^{-6}$	0.64	$1.3x10^{-17}$
CD47[c]	-	-	-0.07	0.39	0.00	0.98
NCR3LG1[d]	-0.34	$3.9 x10^{-5}$	0.15	0.07	-0.26	$1.6x10^{-3}$

[a]CD47-dependence in CD8 T cells was established in WT versus *cd47*$^{-/-}$ mice bearing B16 melanomas (Nath et al. 2019).

[b]Differentially regulated by activation in WT and *cd47*$^{-/-}$ CD8 T cells in vitro (RNAseq data from (Nath et al. 2022))

[c]Differentially expressed in Jurkat WT versus CD47^{-} JinB8 cells (Table S2, Extracted from All_DEGs _pval05_ Data A)

[d]Known MYC-induced gene in melanoma and other cancers (Textor et al. 2016)

Figure S1

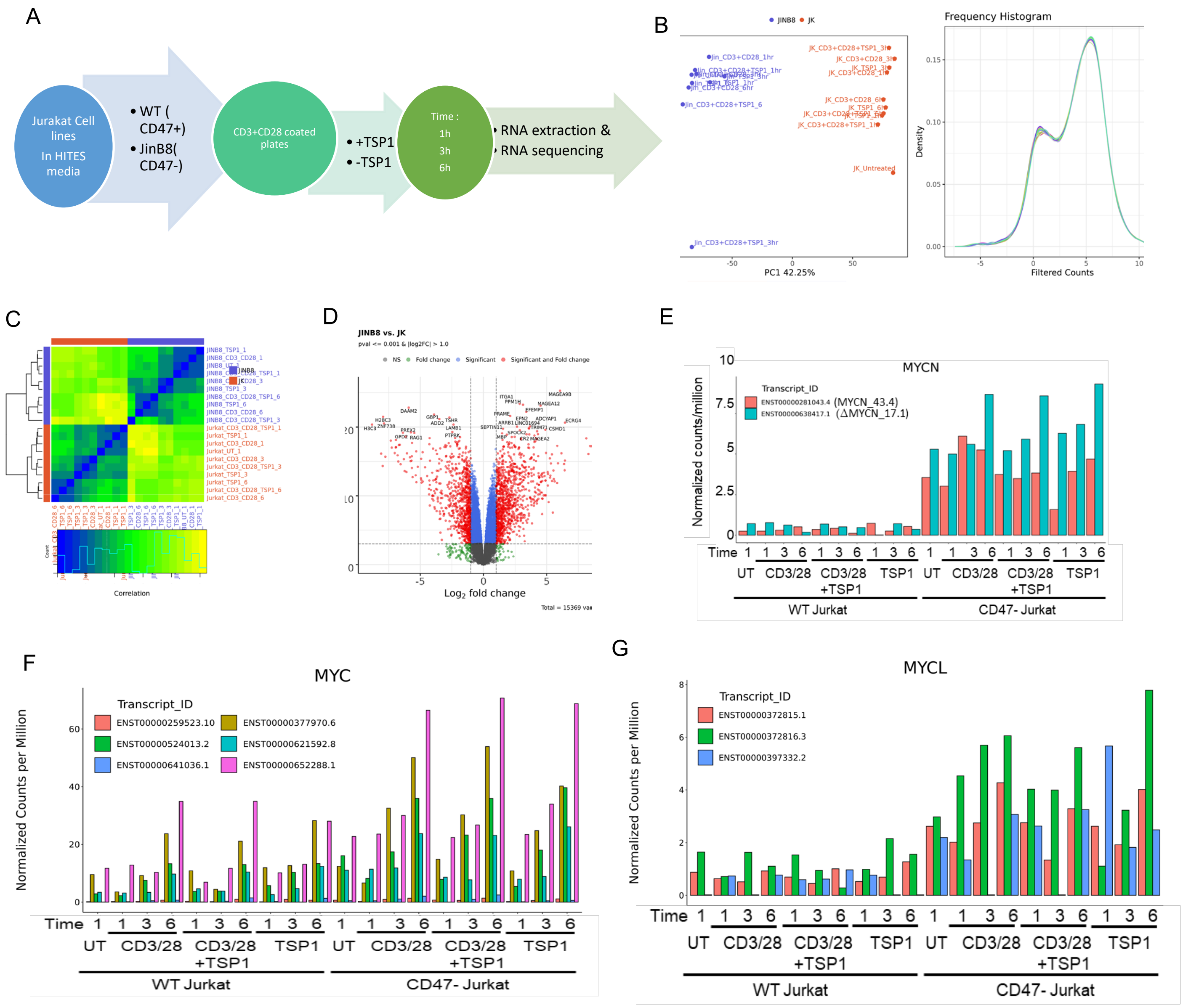
A
Jurakat Cell lines
In HITES media
• WT (CD47+)
• JinB8(CD47-)
CD3+CD28 coated plates
• +TSP1
• -TSP1
Time :
1h
3h
6h
• RNA extraction &
• RNA sequencing
B
JINB8 JK
PC1 42.25%
Frequency Histogram
Density
Filtered Counts
C
Correlation
D
JINB8 vs. JK
Log2 fold change
E
MYCN
Transcript_ID
ENST00000281043.4 (MYCN_43.4)
ENST00000638417.1 (ΔMYCN_17.1)
Normalized counts/million
Time
UT
CD3/28
CD3/28 +TSP1
TSP1
WT Jurkat
CD47- Jurkat
F
MYC
Transcript_ID
ENST00000259523.10
ENST00000377970.6
ENST00000524013.2
ENST00000621592.8
ENST00000641036.1
ENST00000652288.1
Normalized Counts per Million
Time
UT
CD3/28
CD3/28 +TSP1
TSP1
WT Jurkat
CD47- Jurkat
G
MYCL
Transcript_ID
ENST00000372815.1
ENST00000372816.3
ENST00000397332.2
Normalized Counts per Million
Time
UT
CD3/28
CD3/28 +TSP1
TSP1
WT Jurkat
CD47- Jurkat

Figure S2

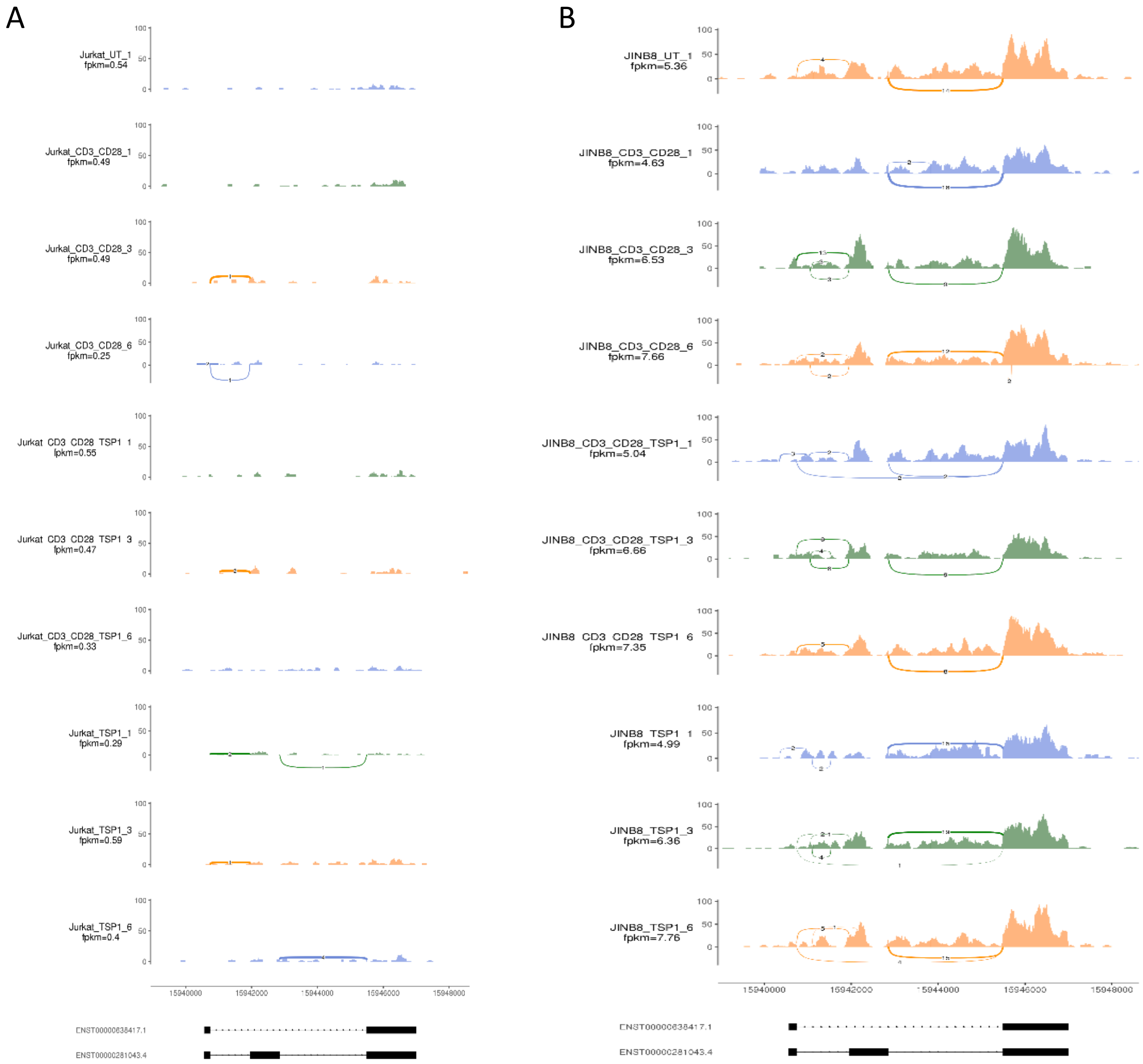
A
Jurkat_UT_1 fpkm=0.54
Jurkat_CD3_CD28_1 fpkm=0.49
Jurkat_CD3_CD28_3 fpkm=0.49
Jurkat_CD3_CD28_6 fpkm=0.25
Jurkat_CD3_CD28_TSP1_1 fpkm=0.55
Jurkat_CD3_CD28_TSP1_3 fpkm=0.47
Jurkat_CD3_CD28_TSP1_6 fpkm=0.33
Jurkat_TSP1_1 fpkm=0.29
Jurkat_TSP1_3 fpkm=0.59
Jurkat_TSP1_6 fpkm=0.4
15940000 15942000 15944000 15946000 15948000
ENST00000638417.1
ENST00000281043.4
B
JINB8_UT_1 fpkm=5.36
JINB8_CD3_CD28_1 fpkm=4.63
JINB8_CD3_CD28_3 fpkm=6.53
JINB8_CD3_CD28_6 fpkm=7.66
JINB8_CD3_CD28_TSP1_1 fpkm=5.04
JINB8_CD3_CD28_TSP1_3 fpkm=6.66
JINB8_CD3_CD28_TSP1_6 fpkm=7.35
JINB8_TSP1_1 fpkm=4.99
JINB8_TSP1_3 fpkm=6.36
JINB8_TSP1_6 fpkm=7.76
15940000 15942000 15944000 15946000 15948000
ENST00000638417.1
ENST00000281043.4

Figure S3

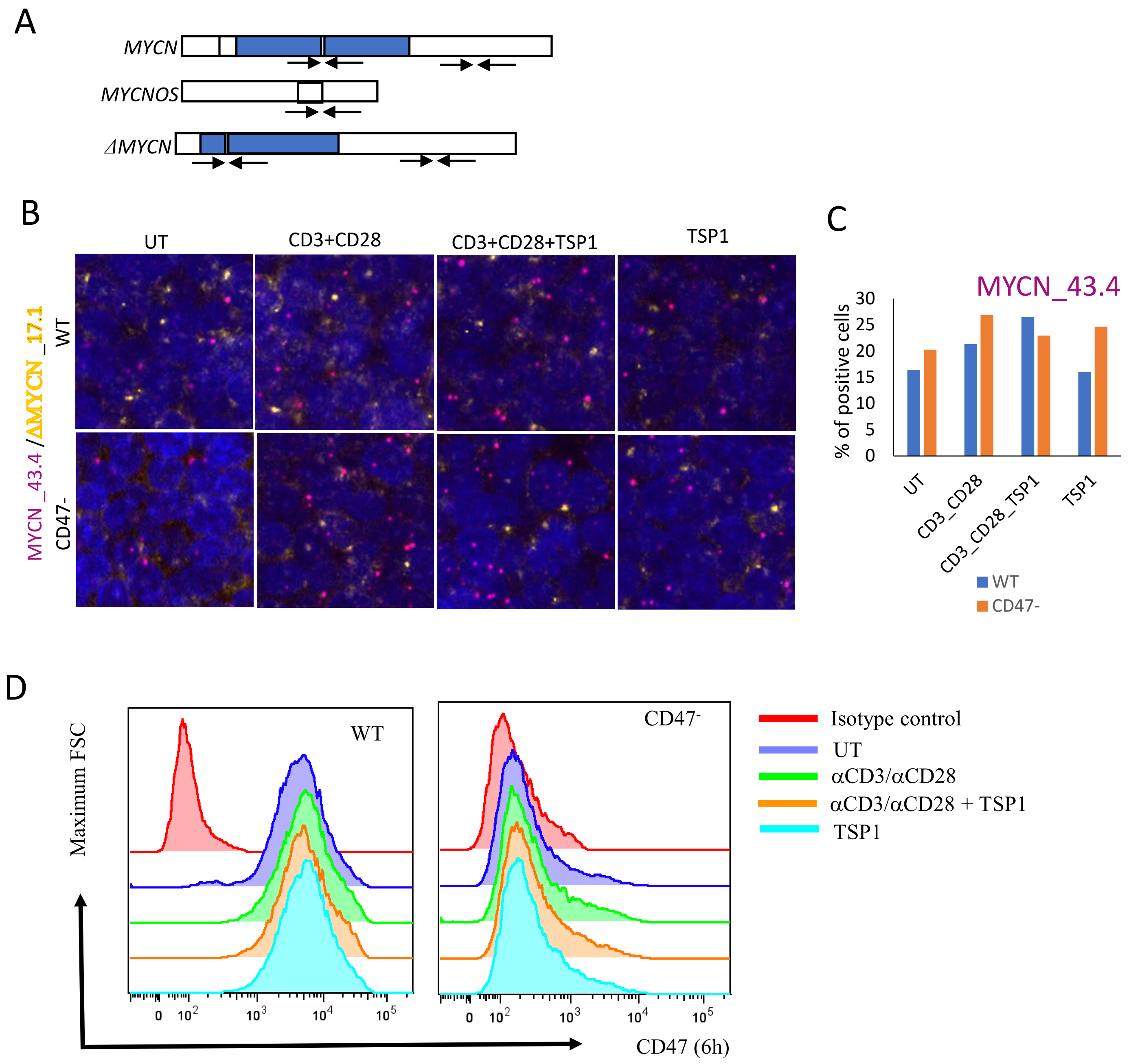

A
MYCN
MYCNOS
ΔMYCN
B
UT
CD3+CD28
CD3+CD28+TSP1
TSP1
MYCN_43.4 / ΔMYCN_17.1
WT
CD47-
C
MYCN_43.4
% of positive cells
30
25
20
15
10
5
0
UT
CD3_CD28
CD3_CD28_TSP1
TSP1
WT
CD47-
D
Maximum FSC
WT
CD47-
Isotype control
UT
αCD3/αCD28
αCD3/αCD28 + TSP1
TSP1
0
10^2
10^3
10^4
10^5
CD47 (6h)

Figure S4

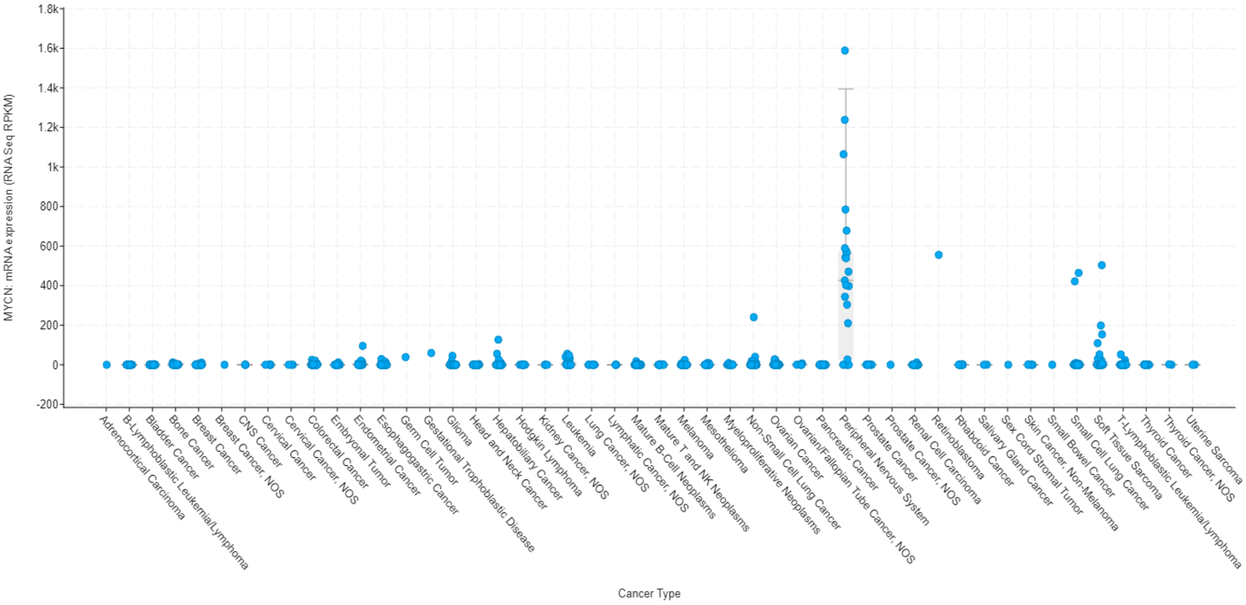

MYCN: mRNA expression (RNA Seq RPKM)
1.8k
1.6k
1.4k
1.2k
1k
800
600
400
200
0
-200
Adrenocortical Carcinoma
B-Lymphoblastic Leukemia/Lymphoma
Bladder Cancer
Bone Cancer
Breast Cancer
Breast Cancer, NOS
CNS Cancer
Cervical Cancer
Cervical Cancer, NOS
Colorectal Cancer
Embryonal Tumor
Endometrial Cancer
Esophagogastric Cancer
Germ Cell Tumor
Gestational Trophoblastic Disease
Glioma
Head and Neck Cancer
Hepatobiliary Cancer
Hodgkin Lymphoma
Kidney Cancer, NOS
Leukemia
Lung Cancer, NOS
Lymphatic Cancer, NOS
Mature B-Cell Neoplasms
Mature T and NK Neoplasms
Melanoma
Mesothelioma
Myeloproliferative Neoplasms
Non-Small Cell Lung Cancer
Ovarian Cancer
Ovarian/Fallopian Tube Cancer, NOS
Pancreatic Cancer
Peripheral Nervous System
Prostate Cancer
Prostate Cancer, NOS
Renal Cell Carcinoma
Retinoblastoma
Rhabdoid Cancer
Salivary Gland Cancer
Sex Cord Stromal Tumor
Skin Cancer, Non-Melanoma
Small Bowel Cancer
Small Cell Lung Cancer
Soft Tissue Sarcoma
T-Lymphoblastic Leukemia/Lymphoma
Thyroid Cancer
Thyroid Cancer, NOS
Uterine Sarcoma
Cancer Type
